# Mouse diet and vendor impact microbiome perturbation and recovery from early-life pulses of amoxicillin

**DOI:** 10.3389/frmbi.2024.1432202

**Published:** 2024-07-29

**Authors:** Noelle Curtis-Joseph, Rachel Peterson, Claire E. Brown, Chapman Beekman, Peter Belenky

**Affiliations:** ^1^ Department of Molecular Biology, Cellular Biology, and Biochemistry, Brown University, Providence, RI, United States; ^2^ Department of Molecular Microbiology and Immunology, Brown University, Providence, RI, United States

**Keywords:** microbiome, gut microbiome, western diet, early-life antibiotics, murine microbiome, antibiotics

## Abstract

The gut microbiome is a dynamic ecosystem shaped by various factors, including diet, sex, and environment. This system plays a crucial role in host health, such that perturbation in the form of antibiotics can lead to a vast array of negative outcomes. Accordingly, a growing body of work seeks to develop interventions to protect the microbiome during antibiotic exposure. While it is well established that antibiotics can disrupt the microbiome in the short term, how the impact of antibiotics is modulated by factors such as diet, sex, and environment is poorly understood. In this study, we analyzed how sex, diet and early life environment (vendor of origin) modulate the impact and recovery of the microbiome in mice treated with oral amoxicillin. Utilizing 16S rRNA gene sequencing and bioinformatic analyses, we looked at the microbiome response to antibiotics under high-sugar and high-fat (Western) and standard high-fiber mouse (Chow) diets in male and female C57BL/6 from Jackson Laboratory, and female mice from Charles River Laboratories. The microbiome composition of each set of mice had a distinct pre-antibiotic starting point, depending on vendor, sex, and diet. These differences were further exacerbated by antibiotic exposure and revealed that each group responded differently to this perturbation. In particular, we found that the Western diet microbiome had an exacerbated response to antibiotics with greater changes in alpha, and beta diversity, and microbial composition when compared to the antibiotic-treated Chow diet cohort. In particular, we detected blooms in *Enterobacteriaceae*, *Streptococcaceae*, and *Peptostreptococcaceae* that were not found in the Chow diet. The response to antibiotics on each diet also appeared to be vendor and sex dependent. Charles River female mice had less *Bifidobacteriaceae*, *Clostridia_UCG.014*, and *Clostridiaceae* compared to Jackson Laboratory females in a Western diet, while female mice had more *Bacteroides*, *Bilophila*, and *Parasutterella* compared to male mice. In a narrow sense, these findings underscore the importance of considering vendor source, diet, and sex when examining antibiotics’ impact on mice. The broader implications suggest that we will likely need to utilize patient-specific microbiome-informed approaches in the development of human therapeutics to safeguard the microbiome during antibiotic exposure.

## 1 Introduction

The gut microbiome is a dynamic ecosystem comprised of thousands of microorganisms that work symbiotically to affect the health of the host organism in part by supporting nutrition and immune system development (Brown et al., 2012). The microbiome assists in digestion and nutrient absorption and synthesizes essential vitamins and amino acids (Ma and Ma, 2019). A diverse gut microbiome supports immune system development and helps maintain intestinal barrier integrity (Thursby and Juge, 2017). Imbalances in microbial composition have been linked to a wide range of conditions including inflammatory bowel disease, obesity, neurological disorders, and more (Maranduba et al., 2015). In turn, this ecosystem is affected by environmental and dietary factors that differentially prime and alter the microbiome in response to adult- or early-life perturbations.

Numerous studies have shown that the composition and function of the early-life microbiome is affected by a range of influences such as mode of delivery, lactation, environmental exposure, diet, and therapeutics (Cox and Blaser, 2015). The early-life microbiome is more susceptible to perturbation because it has a lower diversity than the adult microbiome and is thus more unstable (Guo et al., 2021). For example, a study showed that low-dose penicillin given to dams prenatally shifted early microbial succession and metabolism in pups and caused lasting effects on body composition (Cox et al., 2014). Moreover, some shifts in offspring microbiota induced by maternal antibiotics can persist into adulthood (Cox et al., 2014; Mueller et al., 2015). Disrupted microbial development due to *in utero* antibiotic exposure is associated with increased risk of metabolic disorders and allergic airway disease later in life (Russell et al., 2012; Arrieta et al., 2015).

During development, the microbiome is colonized by microbes that originate from the environment (Ahn et al., 2021). In the laboratory context, the environment manifests as the mouse vendor and the housing facilities. For example, the microbiome composition has been shown to be highly divergent between Janvier Labs, Charles River Laboratories, and Taconic Biosciences mice (Rasmussen et al., 2019). Mice obtained from different vendors harbor distinct microbial profiles that persist even after co-housing (Ericsson et al., 2018; Rosshart et al., 2019). Additionally, vendor-associated microbiome differences can influence disease susceptibility and immune function (Wolff et al., 2020). Overall, these findings highlight the need for researchers to account for vendor-related effects on the microbiome in mouse studies, as differences could impact experimental outcomes and reproducibility.

Sex-mediated differences in the gut microbiota have also been increasingly recognized in both mice and humans. In mice, the gut microbiome differs substantially between males and females, beginning from an early age (Markle et al., 2013; Org et al., 2016). At the phylum level, male mice tend to exhibit decreased *Firmicutes* and increased *Bacteroidetes* compared to females when fed a high fat diet (HFD) (Peng et al., 2020). Within the *Firmicutes* phylum, males have higher levels of *Lachnospiraceae* and *Ruminococcaceae* families, which contain many butyrate-producing bacteria important for intestinal health (Agüero et al., 2016). Furthermore, metagenomic and transcriptomic analyses indicate that male mice show higher potential for branched-chain amino acid biosynthesis while female mice have greater capacity for folate biosynthesis (Peng et al., 2020). Additionally, female microbiomes demonstrate increased glutathione metabolism and antioxidant activity, which may provide protection against oxidative stress (Agüero et al., 2016). Multiple factors may contribute to sex differences in the murine gut microbiome, including hormonal status, immune responses, metabolism, and diet (Markle et al., 2013; Peng et al., 2020).

Diet is another factor that impacts the microbiome. For instance, high-fat diets have been associated with alterations in composition, often linked to metabolic disorders such as obesity and insulin resistance (Caesar et al., 2012; Lam et al., 2015). Conversely, diets rich in fiber have been shown to promote the growth of beneficial bacteria, fostering a more diverse and stable gut microbiota (Singh et al., 2017). Diet also impacts microbial metabolism. For example, diets with varying compositions of fibers, fats, and carbohydrates have been demonstrated to modulate the production of short chain fatty acids (SCFAs), influencing host energy metabolism and immune function (Byrne et al., 2015; Koh et al., 2016). Dietary patterns not only influence the immediate microbial composition but also play a role in shaping the stability and resilience of the microbial community over time (David et al., 2013; Sonnenburg et al., 2016; Costa et al., 2023). Genetic variation in mice has also been shown to modulate responses to dietary interventions, indicating the importance of personalized approaches to decipher diet-microbiome relationships (Org et al., 2015; Kreznar et al., 2017). Moreover, environmental factors, such as specific pathogens or exposure to antibiotics, can interact with dietary influences to shape the overall microbial landscape (Hufeldt et al., 2010; Maurice et al., 2015).

Antibiotic administration has been shown to dramatically impact the composition and diversity of the gut microbiota in mice (Cabral et al., 2020). Even short-term antibiotic treatment can alter the microbiome composition and create long-lasting microbial changes (Dethlefsen et al., 2008; Nobel et al., 2015; Campa et al., 2024). Broad-spectrum antibiotics target gram-positive and gram-negative bacteria and deplete key commensal populations in the murine gut, commonly *Bacteroides*, *Lactobacillus*, and *Bifidobacterium* (Patangia et al., 2022). This depletion causes a reduction in diversity and an overall compositional shift, concurrent with an expansion of *Proteobacteria* and *Verrucomicrobia* (Cho et al., 2012). Beyond these broad signatures, specific antibiotic classes affect the microbiota distinctively based on their mechanisms of action. For example, amoxicillin treatment impacts both gram-negative and -positive bacteria and creates significant shifts in the abundance of *Bacteroidetes* and *Firmicutes* (Lekang et al., 2022).

Antibiotic-induced alterations of the gut microbiome can be long-lasting, often persisting for weeks after cessation of treatment and disrupting colonization resistance—leading to infections such as those caused by *Clostridioides difficile* (Dethlefsen et al., 2011; Collins et al., 2015). Orally administered antibiotics have also been found to hinder and dysregulate early-life formation of the microbiome in neonatal and infant mice (Cox et al., 2014). Even vertically transmitted antibiotics from dams to pups impact microbial succession patterns post-weaning (Miyoshi et al., 2017; Patangia et al., 2022). Furthermore, functional metagenomic analyses show that antibiotics significantly affect metabolism and gene expression of the gut microbiome (Patangia et al., 2022). Microbial pathways linked to nutrient absorption, vitamin synthesis, carbohydrates breakdown, and amino acids synthesis are reduced after antibiotics (Zhang et al., 2012; Nobel et al., 2015). Antibiotic-induced microbiome alterations impair immune system development and exacerbate inflammatory bowel disease in mouse models (Zhang et al., 2012; Nobel et al., 2015). Disruption of the gut microbiome by antibiotic administration can lead to long-lasting changes that may contribute to obesity, highlighting a potential link between antibiotics, gut health, and metabolic disorders (Vallianou et al., 2021). Further research is needed to elucidate the precise mechanisms and implications of antibiotic-induced dysbiosis on obesity development and metabolic health.

Previous work from our group has uncovered compelling evidence highlighting the profound influence of dietary patterns on the interaction between the microbiome and antibiotic response (Cabral et al., 2020). Specifically, our studies have elucidated that Western diet consumption amplifies the microbiome’s susceptibility to antibiotics compared to a control diet regimen, leading to notable alterations in microbial diversity and phylum abundance (Cabral et al., 2020). Furthermore, our findings have shown the exacerbating impact of hyperglycemia in conjunction with antibiotic administration on the susceptibility to microbial infections like *Salmonella*, underscoring the intricate relationship between metabolic health and microbiome resilience (Wurster et al., 2021). Interestingly, our research also reveals the protective role of a high fiber diet in safeguarding essential gut microbes, such as *Bacteroides thetaiotaomicron*, against perturbations induced by the combination of glucose and amoxicillin supplementation, shedding light on the therapeutic potential of dietary interventions in preserving microbial stability and function amid antibiotic exposure (Cabral et al., 2019; Costa et al., 2023; Penumutchu et al., 2023). These discoveries showcase the intricate and multifaceted nature of the gut microbiome’s response to antibiotic perturbations, emphasizing the importance of dietary factors in modulating microbial resilience and health outcomes in antibiotic therapy.

Given the chronic and acute consequences of antibiotic-induced microbiome damage it is essential to understand how antibiotics impact these communities in the short-term and the long-term. Two essential gaps in knowledge include how the initial microbial composition combined with dietary inputs impact antibiotic-induced perturbation. To address this, we treated male and female mice from two different vendors with orally administered amoxicillin on the Standard Chow diet and a Western diet, in four pulses to replicate several rounds of early-life antibiotic exposure. While the antibiotic administration occurred early in life, the antibiotic perturbations persisted for up to 100 days. Overall, we found that both male and female mice from different vendors responded differently to antimicrobials. However, we found that the Western diet negatively impacted the initial perturbation as well as the long-term recovery regardless of sex or vendor.

## 2 Materials and methods

### 2.1 Mouse husbandry

All animals were housed in the Brown University Animal Care Facilities and were approved for use under IACUC protocol number 20-06-0001. C57BL/6 female (FJ) and male (MJ) mice were obtained from Jackson Laboratory, and female (FC) mice were obtained from Charles River Laboratories at 4-weeks-old. Mice were initially separated into two conditions: Standard Chow diet (Laboratory Rodent Diet 5001, LabDiet, St. Louis, MO, USA), and high-fat, high-sugar (Western-style) diet (D12079Bi, Research Diets Inc., New Brunswick, NJ, USA). For each mouse type FJ, MJ, and FC, we set up a total of 20 mice in cages of 5 mice each. In total, 60 mice were used.

**Figure 1:**
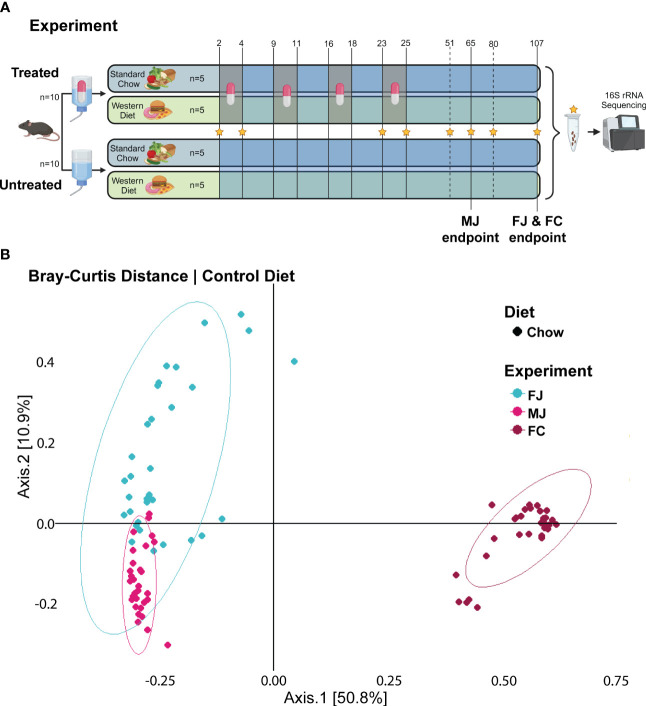
Untreated microbiome composition clusters based on vender and sex. **(A)** Experimental design for female Jackson mice (FJ), male Jackson mice (MJ), and female Charles River mice (FC). Stars indicate the days from which fecal samples were sequenced. Chow diet mice given plain water (Chow -ABX) are the control group in each cohort. Treated mice are indicated by a pill. Untreated mice are indicated by water, alone. **(B)** PCoA plot of Bray-Curtis distance metric of FJ, MJ, and FC mice under control diet.

Starting on day 2, half of the mice from each group were randomly assigned to treatment versus untreated conditions. For the treatment group, 166mg/L amoxicillin was administered via drinking water in 60-hour pulses from days 2-4, 9-11, 16-18, and 23-25. After each 60-hour amoxicillin water pulse, the mice were administered untreated drinking water to replicate several rounds of early-life antibiotic exposure. Following the final antibiotic pulse, the mice were given untreated drinking water for the remainder of the experiment (**Figure 1A**).

### 2.2 Samples collection

Mouse weights were recorded every day from day 0 to 25, then once per week after day 25. Fecal pellets were collected in 1.5mL Eppendorf tubes on days 2 (prior to first antibiotic administration), 4, 23, 25, 51, 65 (MJ only), 80 (FJ and FC only), and 107 (FJ and FC only). Samples were briefly put on ice and then stored at -80°C.

### 2.3 Microbial sample preparation and analysis

Fecal samples were extracted according to the protocol for ZymoBIOMICS DNA Miniprep Kit (Zymo Research, Irvine, CA, USA, Cat #: D4300), and quantified using the Qubit 3.0 Fluorometer. The V4 hypervariable region of the 16S rRNA was amplified using barcoded primers 806R and 515F from the Earth Microbiome Project. Amplicons were made using Phusion High-Fidelity Polymerase (New England Biolabs, Ipswich, MA, USA) and the following cycling conditions: initial denaturation at 98°C for 3 minutes, denaturation for 45 seconds at 98°C, annealing for 1 minute at 50°C, 35 cycles of extension for 1 minute and 30 seconds at 72°C, a final extension for 10 minutes at 72°C, and an infinite hold at 4°C.

Libraries were created by pooling the amplicons for FJ, MJ, and FC into separate pools, and cleaned using the NucleoSpin Gel and PCR Clean-up Kit (Macharey-Nagel, Düren, Germany, Item #:740609.50). Sequencing of the pooled libraries was conducted at the Rhode Island Genomics and Sequencing Center at the University of Rhode Island (Kingston, RI, USA). Samples were paired-end sequenced (2 × 250 bp) using the 500-cycle kit standard protocols on an Illumina MiSeq platform. In total, we sequenced 489 samples with an average sequencing depth of 42083 reads per sample (Weinroth et al., 2022).

### 2.4 Bioinformatic analysis

All raw datasets were first demultiplexed, then filtered and trimmed for quality, checked for chimeras, and denoised using the Divisive Amplicon Denoising Algorithm 2 (DADA2) via the QIIME2 (v.2022.2) pipeline. Trimming removed 10 bases from the 5’ end of the forward and reverse reads, each. Quality filtering removed any samples with a total feature frequency below 1000 reads. Sequences were assigned taxonomic labels (ASV) to the features using the SILVA reference database in the QIIME2 pipeline.

Relative abundance counts and alpha diversity metrics were obtained via QIIME2. Relative abundance significance was calculated using MaAsLin2 (v.1.14.1). Beta diversity metrics were obtained via the R (v.4.3.0) package phyloseq (v.1.44.0). PERMANOVA statistics were assessed using adonis2 from the R package “vegan” (v.2.6.4). Two-Way ANOVA of Shannon alpha diversity was conducted using GraphPad Prism (v.10.1.1). Mann-Whitney testing of Shannon alpha diversity was conducted using the “stats” package in R (v.4.3.0). Differential abundance analysis was conducted using the R package DESeq2 (v.1.40.2).

### 2.5 Availability of data and materials

The datasets containing FASTQ files can be accessed through the BioProject ID PRJNA1126683 on the National Center for Biotechnology Information (NCBI) platform.

### 2.6 Axbio sequencing and data analysis

#### 2.6.1 16S rRNA gene PCR

For the amplification of the V1-V9 region of the 16S rRNA gene, the following primers were used: forward primer (27F1) with anchor sequence 5′-AGRGTTYGATYMTGGCTCAG-3′ and7nbsp;reverse primer (1492R1) with anchor sequence 5′-RGYTACCTTGTTACGACTT-3′ as described in Benjamin J. Callahan et al., 2019 (Callahan et al., 2019). PCR amplification of 16S rRNA gene fragments was conducted using the Super Pfx DNA Polymerase Kit (CWBIO, Taizhou, China) in a total volume of 25 μl containing primer pairs (300 nM each) and 2.5ng of extracted DNA. Amplification was performed with the following PCR conditions: initial denaturation at 98°C for 3 min, 25 cycles of 98°C for 15 s, 62°C for 30 s, and 72°C for 50 s, followed by a final extension at 72°C for 5 min then kept at 4°C infinitely. PCR products were purified using AMPure^®^ XP (Beckman Coulter) beads (1:0.5 sample-to-beads ratio) and quantified with a Qubit^®^ 3.0 Fluorometer (Thermo Fischer Scientific, Waltham, MA, USA).

#### 2.6.2 AxiLona AXP-100 library preparation and sequencing

600ng of purified PCR products were processed using the AxiLona Prep K1.5 Library Preparation Kit (I06, AXBIO, USA, China). End repair, adapter ligation, and single-stranded circularization were performed using the corresponding modules included in the kit. Between steps, purification was carried out using 1X AMPure XP beads (Beckman Coulter) following the manufacturer’s instructions for DNA elution. The constructed libraries were then prepared for sequencing following the detailed steps outlined in the AxiLona Seq 1.0 Sequencing Kit (I08, AXBIO, USA, China). The sequencing complexes, along with other reagents, were loaded sequentially into the sample wells of the gene sequencer cartridge (AX001, AXBIO, USA, China) as per the instructions provided in the manual, and sequencing was performed on the AxiLona AXP-100 Sequencer. AxiLona AXP Sys 1.0 Software was used for data acquisition. AXP sequencing data underwent adapter removal using Cutadapt (version 4.1) with a specified adapter sequence (Martin, 2011). The same size selection criteria were applied as with the nanopore data. A local bacteria database, containing assembly sequences sourced from NCBI (updated to 2023), was downloaded, and then constructed using the script kraken2-build (Wood et al., 2019). Taxonomic assignments were applied to four sets of sequencing data using Kraken2 with default parameters (Wood et al., 2019). Species with a read percentage of less than 1% were further filtered out from the resulting taxonomic identifications. No further read cutoffs were used for the relative abundance analyses.

## 3 Results

### 3.1 Experimental design

To determine how diet, sex, and vendor background influence microbiome composition and response to antibiotics, we fed female Jackson mice (FJ), male Jackson mice (MJ), and female Charles River mice (FC) mice a diet of standard Chow or Western diet, and initially provided plain water to all groups. Starting at 30 days of age, half of each group of mice were designated as the treatment groups and administered amoxicillin via drinking water for 4- 60-hour pulses from days 2 to 4, 9 to 11, 16 to 18, and 23 to 25 to replicate early life antibiotic therapy. Thus, the experimental groups were Chow -ABX (untreated, control group), Chow +ABX (treated Chow), Western -ABX (untreated Western), and Western +ABX (treated Western). Following these amoxicillin pulses, we maintained the mice on plain water to observe microbiome recovery. Specifically, we wanted to see if sex, diet, and vendor impacted the ability of the microbiome to recover to pre-antibiotic composition. We collected fecal pellets on days 2, 4, 23, 25, 51, 65 (MJ only), 80 and 107 (FJ and FC, only) from mouse cohorts, and extracted DNA for 16S amplicon sequencing (**Figure 1A**).

### 3.2 Vendor-specific disparities in baseline microbiome diversity and stability

To perform an initial analysis of the baseline microbiome, we looked at alpha diversity using the Shannon diversity metric and beta diversity using Bray-Curtis distances. At baseline, we found that the FJ and MJ mice were significantly less diverse than the FC mice (**Supplementary Data Sheet 1**). Beta diversity analysis of that same initial time point also indicated that while the FJ and MJ mice were different from each other, the difference between the female mice from different vendors was more dramatic (**Figure 1B**; **Supplementary Table 1**). We then profiled the stability of the microbiome over time in the untreated condition using Bray-Curtis beta diversity to compare day 2 to subsequent days within the same vendor. This analysis indicated that there were significant differences day-to-day and that those differences compounded overtime, likely due to natural variability and the impacts of age on our mice. Interestingly, FJ mice appeared to be more stable than MJ or FC mice, with the smallest number of time points with significant differences in beta diversity relative to day 2 (**Supplementary Table 1**).

**Figure 2:**
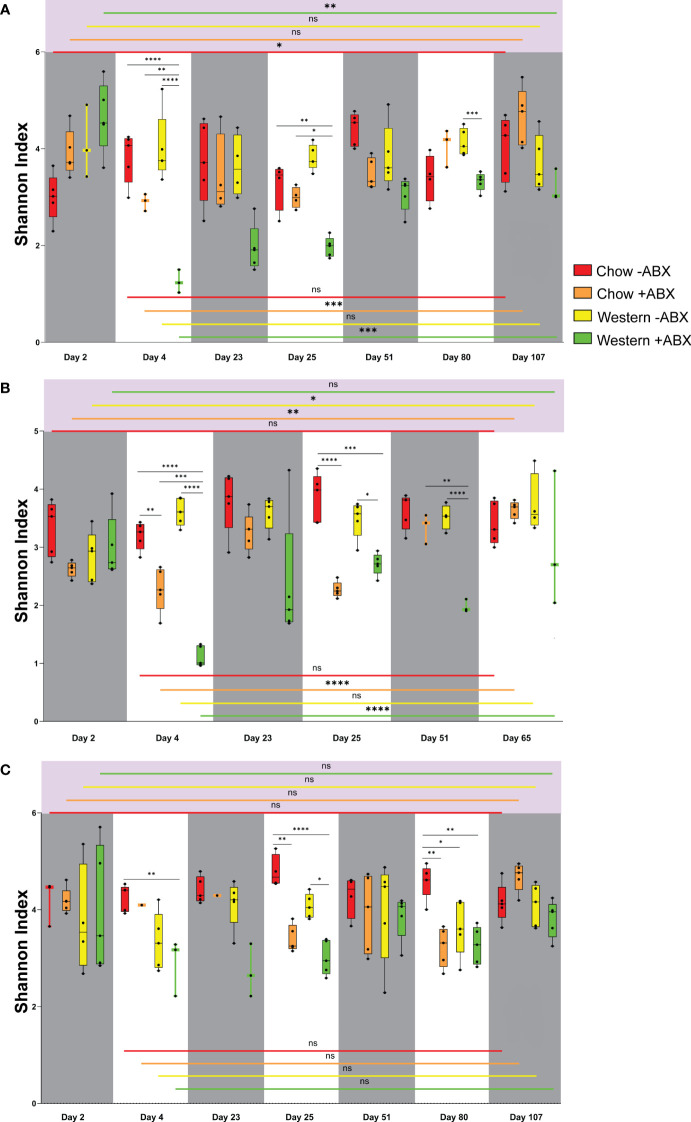
Shannon diversity reveals Chow diet microbial resilience to antibiotics in mice across vendors and sexes. **(A)** Shannon diversity plot for FJ. **(B)** Shannon diversity plot for MJ. **(C)** Shannon diversity plot for FC. Significance values as follows: ns p ≥ 0.05, * p = 0.01 to 0.05, ** p = 0.001 to 0.01, *** p = 0.0001 to 0.001, **** p < 0.0001.

Quantifying alpha diversity of mice on the untreated Chow diet from day 2 through to the terminal points (**Figures 2A, B**), we observed that on day 2, FC untreated Chow diet mice had a higher alpha diversity than both sexes of Jackson mice (FJ p = .0357; MJ p = .0714) (**Supplementary Data Sheet 1**). Alpha diversity in microbiomes of the control diet mice did not show any significant fluctuations when comparing day 2 to the terminal day of the experiment. The highest significance was seen in FJ mice from day 2 to 51 (p = .0229) (**Figure 2A**).

**Figure 3:**
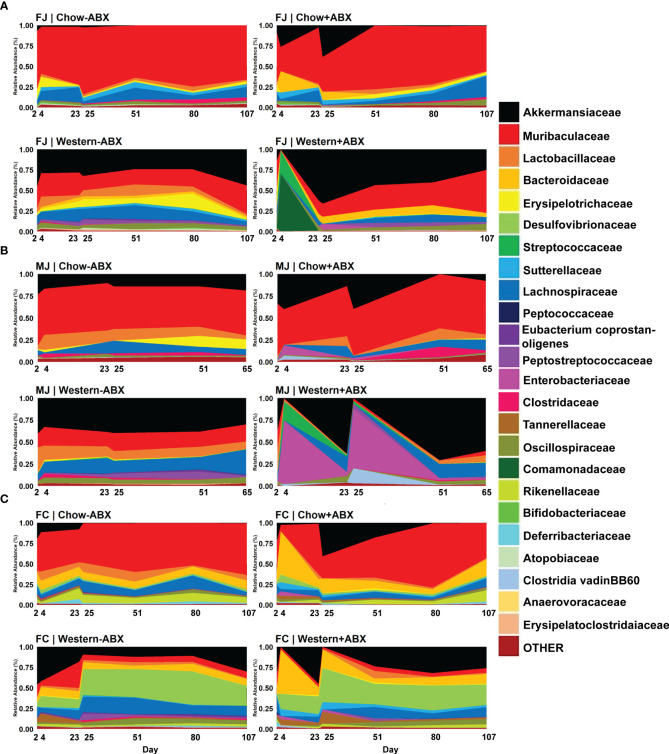
Family-level relative abundance data reveals compositional impacts sustained under respective diets. **(A)** Relative abundance timeseries of FJ. **(B)** Relative abundance timeseries of MJ. **(C)** Relative abundance time series of FC.

As predicted by the beta diversity analysis, we also identified differences in the initial taxonomic composition of each of the communities on day 2. A complete table of relative abundances at various taxonomic levels can be found in **Supplementary Table 2**. To streamline analysis, achieve accurate classification, and to ensure significant taxonomic commonalities between the groups, we focused our differential abundance analyses at the family level. Based on MaAsLin2 analysis, MJ mice had lower *Muribaculaceae* (p-adj = .0504 MaAsLin2) and more *Akkermansiaceae* than FJ mice (p-adj = .0209) (**Figures 3A, B**; **Supplementary Table 3**). FC mice had significantly higher *Bacteroidaceae* (p-adj = .0032), and *Desulfovibrionaceae* (p-adj = .0065), compared to FJ mice (**Figure 3C**). FJ mice had higher *Muribaculaceae* (p-adj = .0424) and *Bacteroidaceae* (p-adj = .0048) than FC or MJ mice, respectively (**Figures 3A–C**). Overall, the baseline composition of each community was quite different, with a large number of differences in individual taxa abundances that can be found in **Supplementary Table 1**. This is consistent with previous studies showing similar vendor differences and indicates that these communities will encounter antibiotic or dietary change from a highly divergent baseline (Rasmussen et al., 2019; Campa et al., 2024).

### 3.3 Antibiotic-treated Chow mice show comparable alpha diversity recovery to untreated Chow but exhibit significant beta diversity changes

Considering the relative stability of the microbiome under a Standard Chow diet, we next examined the effects of antibiotic perturbation in mice from different vendors and sexes. We discovered that the largest perturbations occurred at the conclusion of the antibiotic pulses on days 4 and 25. One of the most dramatic changes was the expansion of *Akkermansiaceae* in all three groups compared to the untreated group, although the change was not significant after every pulse (**Figures 3A–C**; **Supplementary Table 3**). There were also taxonomic changes unique to each group that occurred at each pulse. In FJ mice, there was a bloom of *Enterobacteriaceae* from day 23 to 25 (p-adj = .0413) (**Figure 3A**). MJ experienced a day 4 bloom of *Clostridia vadinBB60* group compared to untreated controls (p-adj = .0071) (**Figure 3B**). In FC mice on day 4, there were blooms of *Bacteroidaceae* (p-adj = .0195), *Sutterellaceae* (p-adj = .0498), and *Gastranaerophilales* (p-adj = .0301) compared to their day 4 control (**Figure 3C**).

Shannon alpha diversity indicated that after the first and second antibiotic pulses, the diversity of FJ and MJ both dropped to remarkably similar levels (**Figures 2A, B**). Furthermore, we observed that of the Jackson mice, only the male mice had significantly decreased alpha diversity after the first antibiotic pulse (p = .0074), which was further exacerbated after the second antibiotic pulse (p < 0.0001) (**Figure 2B**). The second antibiotic pulse in FC mice significantly decreased the microbial diversity (p = .0025) (**Figure 2C**). After concluding the final antibiotic pulse for all mouse groups, the relative abundance plots showed that the microbiome composition changed compared to the control diet cohort (**Figures 3A–C**). This is further confirmed by beta diversity PERMANOVA statistics (**Supplementary Table 1**). A general trend we noticed for all mice is that between the end of the second antibiotic pulse day and the terminal day, there was a complete recovery of the microbiome diversity in treated Chow diet mice, indicating that there is a strong capacity of this community to re-establish homeostasis (**Figures 2A, C**). Thus, while each group had a different response to antibiotics on the Standard Chow, all groups showed a relatively similar capacity to recover in the long-term.

### 3.4 Western diet exacerbates the expansion of inflammatory bacteria

To determine how diet modulates the impact of antibiotics across the different baseline microbiota of FJ, MJ, and FC mice we next compared mice maintained on an untreated Western diet versus the Chow diet. We paid particular attention to the changes occurring on day 25 of both diets and observed visually distinct taxonomic differences in the Western diet compared to Chow, even without antibiotics (**Figures 3A–C**). These differences were also confirmed through MaAsLin2 analysis. For example, in FJ mice there was a significant increase in *Peptostreptococcaceae* (p-adj = .0001), *Akkermansiaceae* (p-adj = .0047), and *Bacteroidaceae* (p-adj = .00047) compared to Chow. MJ mice had an increase in *Erysipelatoclostridaceae* (p-adj = .0071), *Erysipelotrichaceae* (p-adj = .0060), and *Streptococcaceae* (p-adj = .0006). While FC mice had an increase in *Peptostreptococcaceae* (p-adj = .0133), *Desulfovibrionaceae* (p-adj = .0014), and *Clostridaceae* (p-adj = .0100). Conversely, some taxa which significantly decreased in abundance were *Muribaculaceae* (p-adj = .0003) in FJ mice, *Clostridia_UCG.014* (p-adj = .0060) in MJ mice, and *Rikenellaceae* (p-adj = .0030) in FC mice. Thus, Western diet differentially impacts the microbiome of each vendor (**Supplementary Table 1**). In summary, under a Western diet, there is an increase of more gut-inflammatory families (such as Peptostreptococcaceae and Streptococcaceae) primarily in the *Firmicutes* phylum, and a decrease in key SCFA producers such as *Muribaculaceae* and *Clostridia_UCG.014* (Valentina et al., 2017; Grenda et al., 2022).

Under a Western diet, compared to a high fiber (Chow) diet, microbial diversity is expected to decrease due to the lack of microbe-accessible carbohydrates (MAC) (Sonnenburg et al., 2016). However, we found that between the initiation of the Western diet and day 25, there was no significant drop in alpha diversity for any of the mouse groups (**Figures 2A–C**). On the other hand, Bray-Curtis beta diversity PERMANOVA indicated that from days 2 to 25 of the untreated Western diet, microbiome composition is significantly altered after being sustained on the Western diet (**Supplementary Data Sheet 2**). Thus, a Western diet leads to an increased Bray-Curtis beta diversity dissimilarity when compared to the microbiome of those on a Chow diet.

Further analysis with MaAsLin2 found significant associations of the Western diet to the *Peptostreptococcaceae*, *Erysipelatoclostridiaceae*, and *Akkermansiaceae* families. Conversely, Chow diet showed significant associations to the *Clostridia_UCG.014*, *Clostridia_vadinBB60_group2*, and *RF39* families. Interestingly, there were significant differences in families dependent on vendor. For example, in Charles River mice, we observed that under a Western diet, there were significant increases in *Anaerovoracaceae* and *Lactobacillaceae* compared to control diet. However, in Jackson mice there was an increase of *Lachnospiraceae* that was not seen in Charles River mice. When comparing Jackson mice to Charles River mice, we noticed highly significant increases of *Erysipelotrichaceae*, *Atopobiaceae*, and *Akkermansiaceae* in Jackson mice compared to Charles River mice on a Western diet (**Supplementary Table 3**). Thus, not only does diet significantly alter the gut microbial composition, richness, and evenness, but it also does so in a vendor-dependent manner.

### 3.5 Western diet leads to larger antibiotic perturbations than control diet

After establishing the microbiome changes that occur on a Western diet without antibiotics, we then wanted to determine how a Western diet modulates the impact of antibiotics on the microbiome within each cohort. We observed that within each mouse cohort (FJ, MJ, and FC) across all days, Western diet mice treated with antibiotics, compared to treated Chow diet mice, displayed distinct and highly significant microbial compositional differences (Bray-Curtis PERMANOVA, FJ p = 0.001***; MJ p = 0.001***; FC p = 0.001***) (**Supplementary Data Sheet 2**). For all cohorts, antibiotic treatment on Western diet resulted in exacerbated *Akkermansiaceae* blooms compared to treated Chow diet after the final antibiotic pulse (**Figures 3A–C**; **Supplementary Table 3**). The first antibiotic treatment led to an exacerbated initial expansion of *Lactobacillaceae* in Jackson mice on Western diet compared to treated Chow diet mice (FJ p-adj =.0263; MJ p-adj = .0001) (**Figures 3A, B**). Treated Western diet FJ mice also experienced a 70% jump in *Comamonadaceae* (p-adj = .0672) on day 4 compared to treated Chow diet. There was a 28% increase in *Streptococcaceae* (p-adj = .0672) compared to treated Chow (**Figure 3A**). However, FC mice only experienced significant decreases after the first antibiotic pulse in the treated Western diet group with the highest decrease occurring in *Oscillospiraceae* (coef. = -7.9; p-adj = .0212) (**Figure 3C**). Antibiotic treated MJ mice given a Western diet compared to a Chow diet on day 25 had a significantly larger increase in *Enterobacteriaceae* (p-adj = .0008) (**Figure 3B**). Furthermore, day 25 showed a significant increase in *Clostridia vadinBB60* group compared to the treated Chow diet in all Western diet groups (FJ p-adj = .0378; MJ p-adj = .0000; FC p-adj = .0266) (**Figures 3A–C**). Lastly, compared to Charles River mice in the treated Western diet, antibiotics led to an increase of *Bifidobacteriaceae* (p-adj = .0000), *Clostridia_UCG.014* (p-adj = .0001), *Clostridiaceae* (p-adj = .0001) in Jackson mice (**Figures 3A–C**).

To further examine diet-dependent impacts of antibiotics on microbial composition in each vendor cohort, we calculated the effect size of antibiotic treatment within each diet based on average beta diversity distances between untreated and antibiotic-treated mice. We observed that in Jackson mice, the highest changes in microbiome composition were after the second antibiotic pulse, and the effect size in Western diet mice was higher than Chow (**Supplementary Data Sheet 3**; **Supplementary Table 4**). Conversely, in FC mice, the effect size in Chow diet mice was higher than in the Western diet after the second antibiotic pulse (**Supplementary Data Sheet 3**). For all vendor cohorts, the effect size of Western diet was higher compared to Chow diet on day 4. Thus, Western diet with antibiotic insult differentially impacts microbial response and recovery dependent on sex and vendor.

### 3.6 Chow diet maintains microbiome diversity despite antibiotic challenges; Western diet leads to significant antibiotic-induced dysbiosis

**Figure 4:**
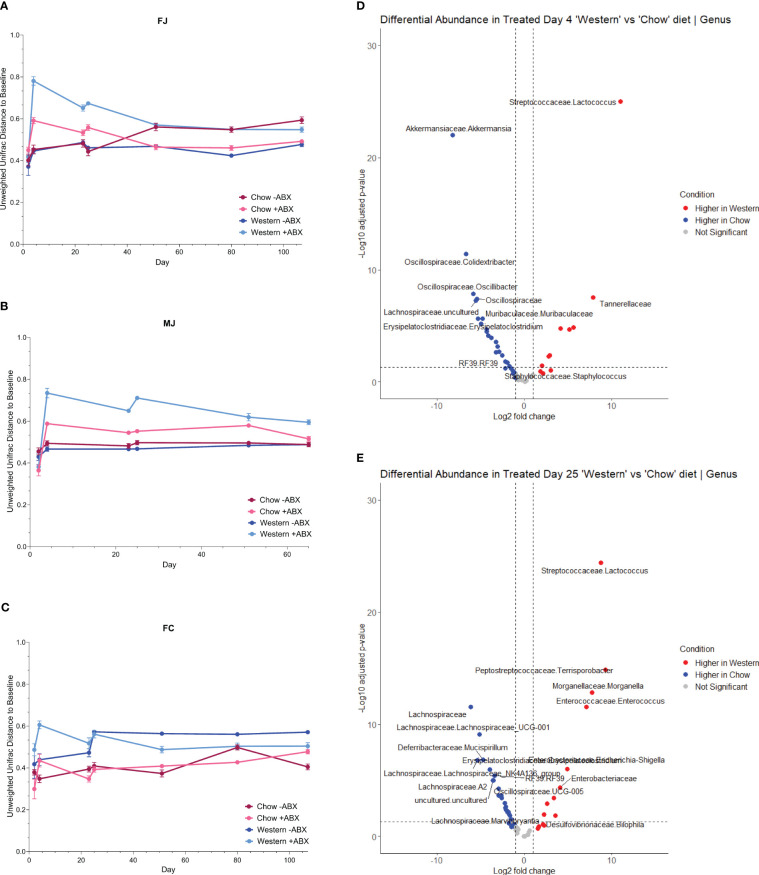
Antibiotics are more disruptive to the microbiome with a Western diet. **(A)** FJ Unifrac distances relative to baseline (day2) for each diet. Error bars are SEM. **(B)** MJ Unifrac distances relative to baseline for each diet. Error bars are SEM. **(C)** FC Unifrac distance to baseline for each diet. Error bars are SEM. **(D)** Genus-level DESeq2 differential abundance of day 4 mice from all cohorts. Comparing Western +ABX to Chow +ABX. **(E)** Genus-level DESeq2 differential abundance of day 25 mice from all cohorts. Comparing Western +ABX to Chow +ABX.

We then looked at time-dependent changes in Unweighted Unifrac distance from baseline composition (day2) to determine community shifts over time. We observed that Western -ABX was stable, in both Jackson groups (**Figures 4A, B**). In MJ mice, Chow and Western diet alone also did not appear to have a significant impact in the Unifrac baseline microbiome (**Figure 4B**). In both FJ and MJ mice, we observed that antibiotics had a greater impact on community stability on the Western diet compared to the Chow diet. This indicates that the Chow diet promotes stability whereas the Western diet induces greater microbiome variability after antibiotic exposure. Conversely, in FC mice we found that antibiotics had a relatively low impact on community stability in either the Western or the Chow diet. It is possible, however, that the antibiotic related disruption is masked by the large change in beta diversity that is driven by introduction of the Western diet (**Figure 4C**). Thus, in both Jackson cohorts, the antibiotics destabilized the microbiome more than diet. However, we noticed that in the Western diet, the long-term recovery of the microbiome was not as complete compared to Chow (**Figures 4A, B**).

The higher shifts in community composition after the first antibiotic pulse within mice on a Western diet compared to Chow diet further reinforced that diet and antibiotics differentially impact the microbiome (**Figure 4D**). Next, we used MaAsLin2 to identify taxa associated with antibiotics, and diet. We observed that from the first antibiotic time point to the terminal timepoint, antibiotics across all mouse cohorts are strongly associated with *Enterobacteriaceae* (p-adj = .0000), *Acholeplasmataceae* (p-adj = .0004), and *Clostridia_vadinBB60_group2* (p-adj = .0027) (**Figure 4E**; **Supplementary Table 3**). With the additional caveat of diet, we discovered that antibiotics in Western diet compared to a Chow diet, are strongly associated with *Enterobacteriaceae* (p-adj = .0000), *Streptococcaceae* (p-adj = .0000), and *Peptostreptococcaceae* (p-adj = .0000) (**Supplementary Table 3**).

### 3.7 Western-diet differentially affects the microbial composition of female mice compared to male mice when given antibiotics

Research has shown that male and female mice have a sex dependent microbiome composition (Campa et al., 2024). We utilized DESeq2 and MaAsLin2 to determine the sex dependent microbial changes incurred under antibiotic administration in Western diet mice. DESeq2 indicated that on all days, compared to female mice, male mice had more *Anaeroplasma*, *Terrisporobacter*, and *Clostridium_sensu-stricto_13* while females had more *Bacteroides*, *Bilophila*, and *Parasutterella* than males (**Supplementary Data Sheet 4**). MaAsLin2 indicated that in females, the bacteria most significantly associated with Western diet were *Bacteroidaceae* (p-adj = .0000), *Tannerellaceae* (p-adj = .0000), and *Muribaculaceae* (p-adj = .0000). While in males they were *Streptococcaceae* (p-adj = .0000), *Lactobacillaceae* (p-adj = .0002), and *Enterobacteriaceae* (p-adj = .0000) (**Supplementary Data Sheet 4**). The Bray-Curtis dissimilarity measurements for FC, FJ, and MJ mice at the two antibiotic pulse points and on the final day showed that female mice on days 4 and 25 demonstrated an overlap between the Chow diet groups, regardless of antibiotic treatment, as well as with the Western diet. In contrast, males on the Western diet with antibiotic treatment were completely distinct from all other diet groups throughout the antibiotic pulse periods (**Supplementary Data Sheet 4**). Indicating that the microbiome of male mice may be more vulnerable to antibiotic induced alterations while on a Western diet and undergoes more significant long-term compositional changes than that of female mice receiving antibiotics on a western diet.

### 3.8 High-resolution sequencing highlighted gut microbiome changes pre- and post-antibiotics, revealing intricate dietary responses

**Figure 5:**
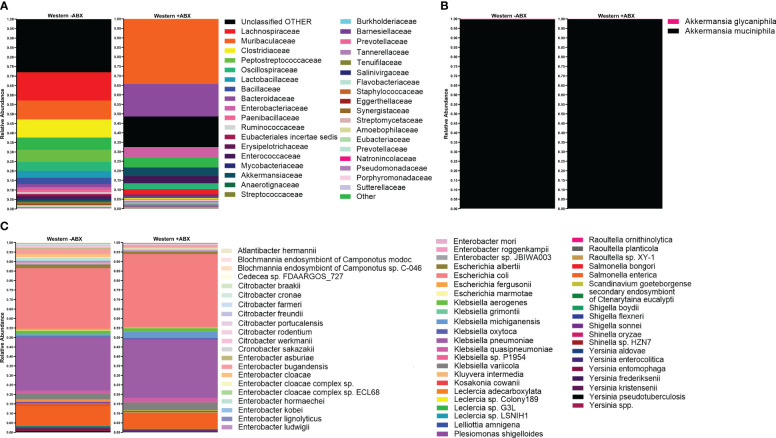
Species-level sequencing highlighted gut microbiome changes pre- and post-antibiotics, revealing intricate dietary responses. **(A)** Top 30 most abundant families in Western -ABX and Western +ABX using the AXP100 platform. **(B)** AXP100 sequencing species-level relative abundance in the Akkermansiaceae family in the Western -ABX and Western +ABX groups. **(C)** AXP100 sequencing species-level relative abundance in the Enterobacteriaceae family in the Western -ABX and Western +ABX groups.

The analysis conducted so far utilized short-read 16S sequencing and, as a result, was not able to determine composition down to the species level. An alternative to the short-read paradigm is a new expansion of long-read technologies. Here, we trialed a previously untested technology provided by Axbio. Axbio’s AXP100 utilizes a sequencing-by-synthesis approach paired with electrical current measuring nanopores. The potential of this technology is that it can produce full-length 16S rRNA reads with acceptable Q scores. This part of the project was aimed at trialing this new technology and determining species identity of differentially abundant taxa induced by antibiotics on the Western diet in FJ mice. As this platform was in development, we were limited to testing 11 samples due to instrument availability, and we do not view this as a complete platform validation, but rather as an effective test run. We first used AXP100 sequencing to get a community snapshot of the top 30 most abundant families in the antibiotic-treated and untreated groups at the 25-day time point from the total 11 samples. Working with the 1 untreated and 3 treated samples comprising this day 25 time point, we looked at families that were significantly expanded during antibiotic perturbation in our Illumina data (**Figure 5A**). Particularly, we observed increases in *Enterobacteriaceae* and *Akkermansiaceae* in the treated condition. To determine the specific taxonomic changes that occurred under antibiotic treatment, we then looked at the species-level breakdown within each of these families in the Axbio dataset (**Supplementary Table 5**). In *Akkermansiaceae*, we observed a total of two species, *Akkermansia muciniphila* and *Akkermansia glycaniphila* (**Figure 5B**). We observed that *A. glycaniphila* decreased and *A*. *muciniphila* increased after antibiotic treatment. Of the 63 species we observed in the *Enterobacteriaceae* family, some of the most abundant species were *Escherichia coli*, *Klebsiella michiganensis*, *Klebsiella oxytoca*, and *Klebsiella quasipneumoniae* (**Figure 5C**). While this analysis did not have sufficient n to conduct statistical analysis, it did demonstrate that Axbio AXP100 long-read sequencing can be used to conduct species-level analysis.

## 4 Discussion

In this study, we explored the impact of diet and antibiotic treatment on the gut microbiome of male and female mice sourced from Jackson Laboratory and female mice from Charles River Laboratories. The four pulses of antibiotic treatment were concluded on day 25, after which we closely monitored the recovery of the gut microbiome. Here, we showed that diet impacts the microbiome response to antibiotics, with a Western diet having a more detrimental effect on the alpha and beta diversity than a Chow diet. We further showed that sex and vendor differentially shape the initial microbiome and are associated with the initial microbiomes response to diet- and antibiotics.

In addition to the data compiled in the results section, this work also gave us the opportunity to monitor the impact of early life antibiotic exposure on weight. We weighed the mice throughout the study period and plotted the data in **Supplementary Data Sheet 5**. Interestingly, we found a highly divergent impact of antibiotic exposure on animal weight. In particular FJ mice, on both the Western and the Chow diet, gained less weight after antibiotic exposure compared to their untreated counterparts. However, this result was only significant in the Western group (**Supplementary Data Sheet 5**). In MJ and FC mice, antibiotic exposure did not have a significant impact on weight. The induction of weight gain by beta-lactam antibiotics has long been observed and utilized in agricultural livestock (Weber et al., 1952; Hill, 1953; Angelakis, 2017). A robust body of work links this weight gain to microbiome modulation impacting nutrient extraction, inflammation, or food consumption (Allen et al., 2014). It is proposed that orally administered antibiotics eliminate anaerobes in the gut, and increase the obesogenic, facultative anaerobes such as *Enterobacteriaceae* (Rizzatti et al., 2017). As a result, there is a change in microbe-derived metabolites, and reduced turnover of the gut mucosa—all of which may contribute to weight gain (Anderson et al., 1999). However, work from Cox and Blaser showed that the host does not gain weight when the antibiotics are administered at therapeutic levels, but rather at subtherapeutic levels (Cho et al., 2012; Cox and Blaser, 2015). Our results in conjunction with current literature point out that antibiotic exposure does not have a universal obesogenic result, and that differences in microbiome response may also lead to differential obesogenic impacts (Leong et al., 2020; Wan et al., 2020).

As discussed above, our results indicated that not only did our mice have different microbiome compositions based on vendor, but also that the baseline composition of each mouse group was associated with a different response to diet and to antibiotics. Other studies have demonstrated that the abundance of *Akkermansia*, which we found in higher quantities in Jackson mice, has been found to impact a large number of fecal-derived metabolites (Visconti et al., 2019). *Akkermansia* are also known to be recalcitrant to various antibiotics (de Nies et al., 2022). Thus, the observed bloom in *Akkermansia* after antibiotic treatment is predictable based on its antibiotic recalcitrance and likely to have downstream impacts on microbial composition due to the large number of metabolites that it produces. Furthermore, we demonstrated that dietary composition modulates the gut microbiome’s response to antibiotic exposure. Mice on a Chow diet retained alpha diversity, and had an overall lower, beta diversity perturbation compared to their Western diet counterparts during and after antibiotic exposure. This reduced microbial stability after antibiotic exposure to the Western diet was mediated by multiple taxa, but in particular, we found that *Akkermansiaceae* and *Enterobacteriaceae* proliferated in Western diet-fed, antibiotic-treated mice. Other research has shown that *Akkermansiaceae* and *Enterobacteriaceae* both increase in Western diet and in antibiotic conditions (Dudek-Wicher et al., 2018; Yip et al., 2023). *Akkermansiaceae* is linked to mucin degradation and potentially heightened susceptibility to enteric pathogens, and has been shown to be more prevalent in Western diet. Some findings indicate a decline in *Akkermansiaceae* following Western diet, and antibiotic treatment, a decline that is also associated with obesity (Cani and de Vos, 2017; Choi et al., 2021). Some work proposes that diets low in fiber can promote mucin consumption on the G.I. epithelium by muciniphilic bacteria, and as a result, promote the proliferation of pathogenic bacteria (Everard et al., 2013). Other studies have found that *Verrucomicrobia* blooms post-Western diet and antibiotics due to a decrease in the enzymes that aide in polysaccharide breakdown (Dubourg et al., 2013; Dao et al., 2016; Cabral et al., 2020). While some work shows that *Akkermansia* is potentially detrimental to microbiome homeostasis, many other studies postulate that it is actually a probiotic promoting many aspects of host health (Jian et al., 2023). It is likely that this microbiome has a context-specific role in host health that is defined by microbiome members and dietary conditions (Ahn et al., 2020; Bourdeau-Julien et al., 2023).

As previously stated, we observed an increase in *Enterobacteriaceae* in Western-diet fed, antibiotic-treated mice. Typically, an expansion of *Enterobacteriaceae* is a potent marker of gut dysbiosis and is associated with further increase of other bacteria in the *Proteobacteria* phylum (Byndloss et al., 2017; An et al., 2021). It is believed that the expansion of *Enterobacteriaceae* in the gut is correlated with an increase of nitrate and oxygen production from the host that results from perturbations such as a high-fat, high-sugar diet, and antibiotics (Baldelli et al., 2021). Under the aforementioned conditions, the microbiome can experience further increases in inflammatory bacteria that can lead to intestinal diseases such as Ulcerative Colitis and Crohn’s Disease (Winter et al., 2013; Tiso and Schechter, 2015).

While *Enterobacteriaceae* can act as both commensals and pathogens, they make up less than 1% of a typical healthy microbiota (Martinson et al., 2019; Gouveia et al., 2024). During disease, dietary perturbation, or antibiotic treatment the proportion of these bacteria can climb to much higher levels; a condition that is associated with pathogenesis. In addition, taxa such as Salmonella can be directly pathogenic and invasive through the G.I (Zhang et al., 2009; Martinson et al., 2019). Conditions that reduce microbiome diversity or promote G.I. inflammation can trigger this bloom and pathogenesis. This leads to an overall imbalance in the gut microbial community and the nutrient extraction capacity of the microbiota. High levels of *Enterobacteriaceae* are associated with obesity in humans (Santacruz et al., 2010; Gouveia et al., 2024).

Enterobacteriaceae bloomed after antibiotics in both the Western and Chow conditions; however, in the Chow diet the magnitude of the bloom was smaller and was possibly mitigated by an unexpected proliferation of *Firmicutes*. Firmicutes are a bacterial phylum known for SCFA production and potential anti-inflammatory properties (Sokol et al., 2008). This suggests that although antibiotic perturbation disrupts the microbiome, favoring a rise in inflammatory bacteria, a high fiber diet appears to have the potential to mitigate these microbial imbalances induced by antibiotic treatment. These findings highlight the importance of diet on the resilience of the gut microbiome to antibiotic perturbations and potential health consequences for the host.

On a subset of samples, we conducted long-read species-level analysis on the Axbio AXP100 sequencing platform. This analysis detected species from a total of 368 families. Whereas the Illumina platform detected a total of 68 families in the full dataset, but only 35 families in the smaller sample set profiled by both platforms. We attribute the discrepancies that we observed in the family-level identifications to the longer read length, and therefore, heightened discrimination between closely related families with reduced error (Amarasinghe et al., 2020; Kim et al., 2024). As stated above, the benefit of the Axbio platform is its ability to achieve species-level identification. Looking at families determined to be statistically different between Western -ABX and Western +ABX on the Illumina platform, we were able to identify multiple species from the *Enterobacteriaceae* family. In particular, *K. michiganensis*, *E. coli*, *K. quasipneumoniae*, and *K. oxytoca*, are noteworthy due to their association with HFD. Similar changes have been detected in humans and mice fed a HFD (Agus et al., 2016; Faqerah et al., 2023). However less work has been done in associating antibiotic exposure to a bloom of these microbes in the context of different diets. Our work highlights that not only do these species bloom under Western diet conditions but that their bloom is exacerbated by antibiotic exposure. Understanding why these species bloom is important, as each species has multiple associated morbidities ranging from infection to auto-brewery syndrome (Yuan et al., 2019). The quick replication time of *E. coli* and other *Enterobacteriaceae* may explain their bloom after antibiotic-induced microbiome disruption (Han et al., 2024). Additionally, the low-MAC environment of the Western diet may further support their bloom (Looft and Allen, 2012). Furthermore, many *Klebsiella* spp. have robust beta-lactam resistance repertoires providing them an additional advantage in the antibiotic treated community (Korry et al., 2020; Gaballah et al., 2023).

Whereas species-level analysis of the *Enterobacteriaceae* family revealed a multitude of unique species, the same analysis of the *Akkermansiaceae* family revealed only two, *A. muciniphila* and *A. glycaniphila*. Since these muciniphilic bacteria are important to host physiology and stability (Si et al., 2022), defining which species populate our mice is essential. In particular, most studies have focused on *A. muciniphila*, whereas fewer have discussed *A. glycaniphila*, and knowing that both occupy the murine niche may change our understanding of mucin-degrading activity in the G.I.

## 5 Limitations

Overall, this work contributes to understanding how diet influences the gut microbiome’s response to antibiotics. Nevertheless, it is limited by experiment duration and sample size. While we utilized multiple cages in order to define cage effect, we only sampled mice from a specific vendor at one time point. This is a potential limitation as the baseline composition from each vendor may change over time and this was not assessed by our study. In addition, not all mice groups were observed for the same duration; for instance, male mice from Jackson Laboratory were not monitored up to 107 days. The endpoints were selected based on a plateau of mouse weights, as this was the initial goal of the study. However, a longer-term study could offer further insight into the lasting effects of antibiotic exposure and low-MAC diets. Besides duration, diet composition is an additional factor that was not fully explored in this work as there are many ways to achieve a high-fat high-sugar diet and each of those permutations would have a differing impact on the microbiome. In addition, sex as a variable was not exhaustively explored. Female Charles River mice were used as a comparison to the female Jackson mice, as a way to determine vendor impact. However, further sex comparison studies, including male mice from Charles River Laboratories, would enhance our understanding of sex-related microbiome variations. We also utilized a single dose of one antibiotic delivered via drinking water, additional concentrations, modes of delivery, or antibiotic types may have vastly different results. Future studies should consider these factors, alongside dietary interventions, and pre- and probiotic therapies, to develop strategies that mitigate complications associated with antibiotics and promote microbiome recovery, taking into account the host’s sex, environmental influences, and disease predisposition. While our findings emphasize the interaction between diet, environment, and biological attributes, like sex, in determining the gut microbiome’s adaptive capacity and ultimate return to a state of equilibrium post-antibiotic exposure, human-based studies would be needed to fully define these factors.

## Data availability statement

The datasets presented in this study can be found in online repositories. The names of the repository/repositories and accession number(s) can be found in the article/**Supplementary Material**.

## Ethics statement

The animal study was approved by Brown University Institutional Animal Care and Use Committee (IACUC). The study was conducted in accordance with the local legislation and institutional requirements.

## Author contributions

NC-J: Conceptualization, Data curation, Formal analysis, Investigation, Methodology, Project administration, Resources, Software, Supervision, Validation, Visualization, Writing – original draft, Writing – review & editing. RP: Conceptualization, Writing – review & editing, Data curation, Project administration, Investigation. CEB: Conceptualization, Data curation, Formal analysis, Investigation, Methodology, Writing – review & editing. CB: Conceptualization, Supervision, Writing – review & editing, Data curation. PB: Conceptualization, Supervision, Writing – review & editing, Funding acquisition, Methodology, Formal analysis, Investigation, Project administration, Resources.
