# Supplementary material for: Mouse diet and vendor impact microbiome perturbation and recovery from early-life pulses of amoxicillin
Source: Front Microbiomes. 2024 Jul 29;3:1432202. doi: 10.3389/frmbi.2024.1432202 (PMC12993551; doi:10.3389/frmbi.2024.1432202)
Supplement: Supplementary file 1 [file DataSheet_1.docx]

**Supplementary Data Sheet 1 – Shannon Diversity**

**Supplementary Figure S1** – FJ vs MJ p = .4206 ns, FJ vs FC p = .0357*, MJ vs FC p = .0714. Mann-Whitney calculated using R package “stats” (v.4.3.0). [ns p ≥ 0.05, * p = 0.01 to 0.05, ** p = 0.001 to 0.01, *** p = 0.0001 to 0.001, **** p < 0.0001]
