# Supplementary material for: Mouse diet and vendor impact microbiome perturbation and recovery from early-life pulses of amoxicillin
Source: Front Microbiomes. 2024 Jul 29;3:1432202. doi: 10.3389/frmbi.2024.1432202 (PMC12993551; doi:10.3389/frmbi.2024.1432202)
Supplement: Supplementary file 2 [file DataSheet_2.docx]

**Supplementary Data Sheet 2 – Bray-Curtis**


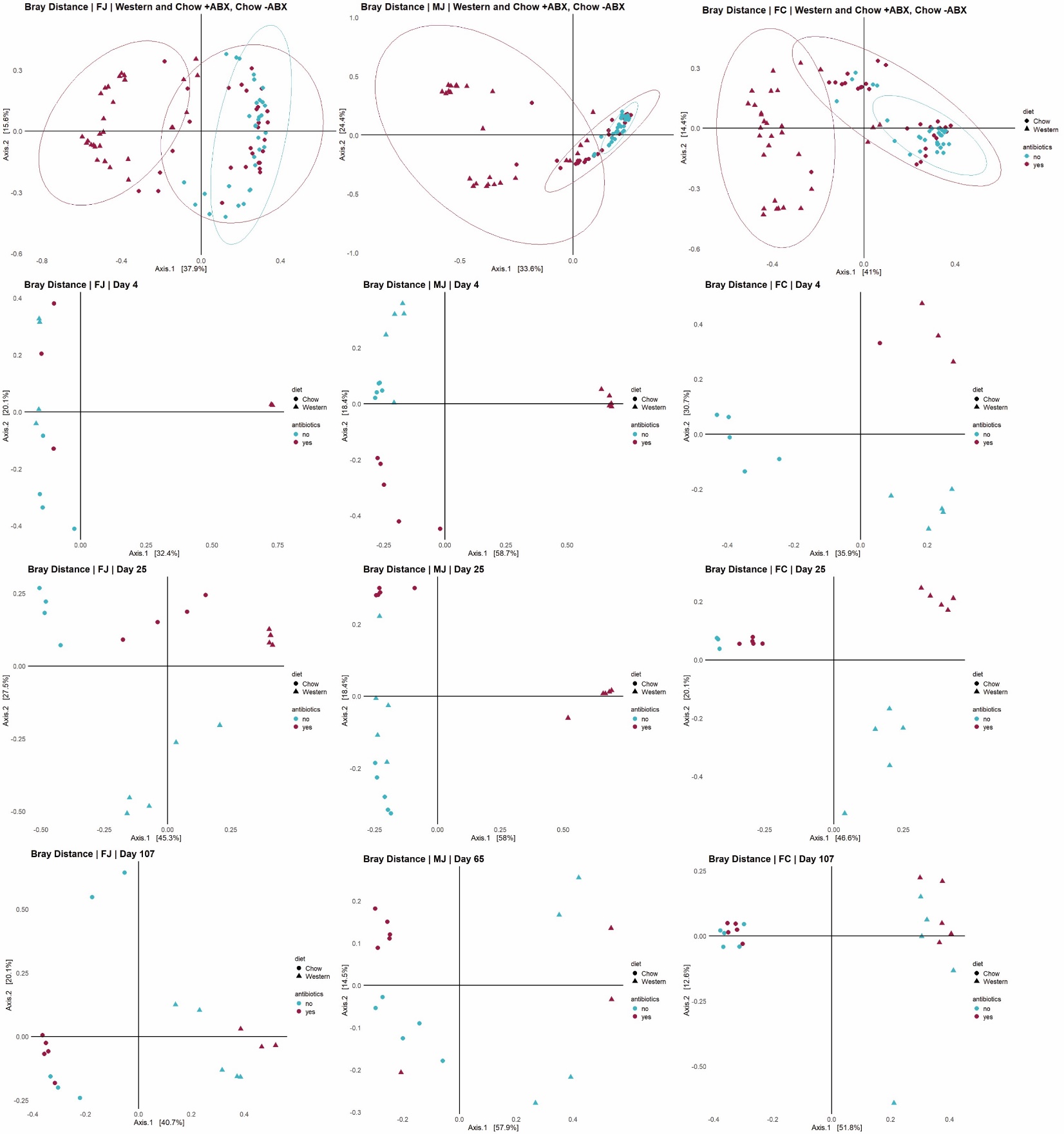


**Supplementary Figure S2** – Bray-Curtis plots of FJ, MJ, and FC cohorts under various diet conditions and days.
