# Supplementary material for: Mouse diet and vendor impact microbiome perturbation and recovery from early-life pulses of amoxicillin
Source: Front Microbiomes. 2024 Jul 29;3:1432202. doi: 10.3389/frmbi.2024.1432202 (PMC12993551; doi:10.3389/frmbi.2024.1432202)
Supplement: Supplementary file 3 [file DataSheet_3.docx]

**Supplementary Data Sheet 3 – Effect Size**


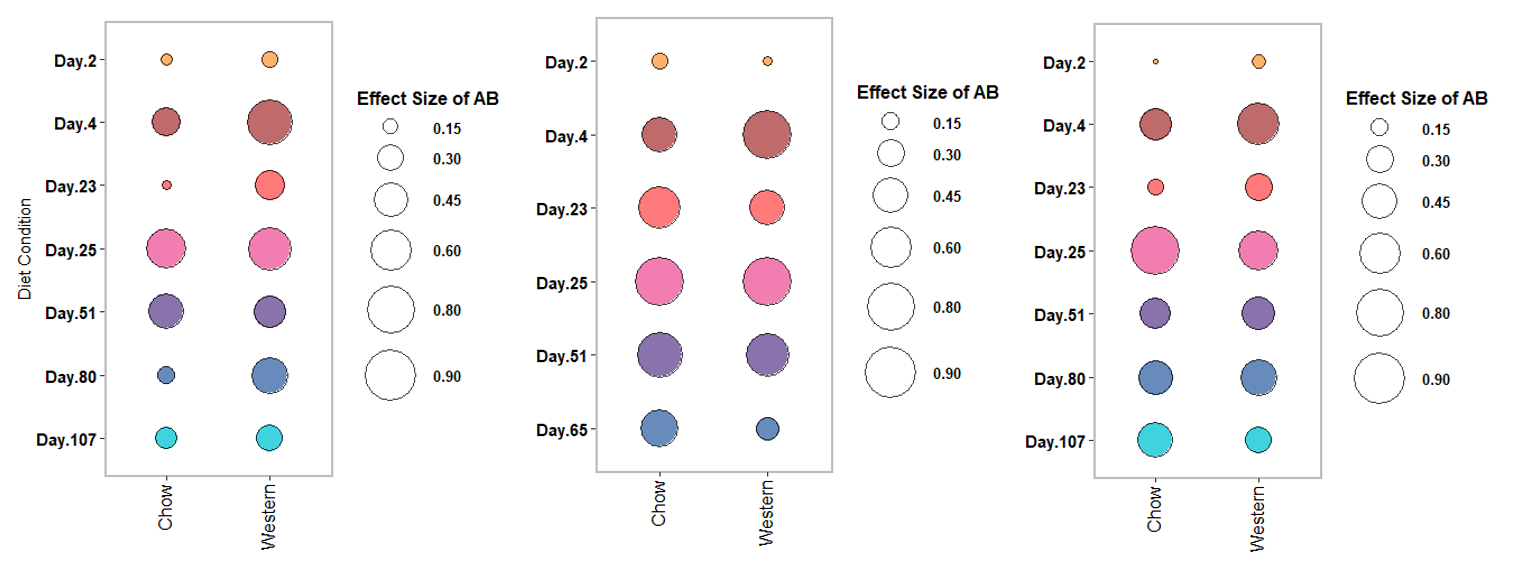


**Supplementary Figure S3 – A)** Effect size bubble plot of microbiome composition changes between untreated diet and treated diet conditions for FJ calculated using the Bray-Curtis PERMANOVA from the vegan R package (v. 2.6-4). **B)** Effect size bubble plot of microbiome composition change between untreated diet and treated diet conditions for MJ calculated using PERMANOVA in the vegan R package (v. 2.6-4). **C)** Effect size bubble plot of microbiome composition change between untreated diet and treated diet conditions for FC calculated using PERMANOVA from the vegan R package (v. 2.6-4).
