# Supplementary material for: Mouse diet and vendor impact microbiome perturbation and recovery from early-life pulses of amoxicillin
Source: Front Microbiomes. 2024 Jul 29;3:1432202. doi: 10.3389/frmbi.2024.1432202 (PMC12993551; doi:10.3389/frmbi.2024.1432202)
Supplement: Supplementary file 4 [file DataSheet_4.docx]

**Supplementary Data Sheet 4 – DESeq2**


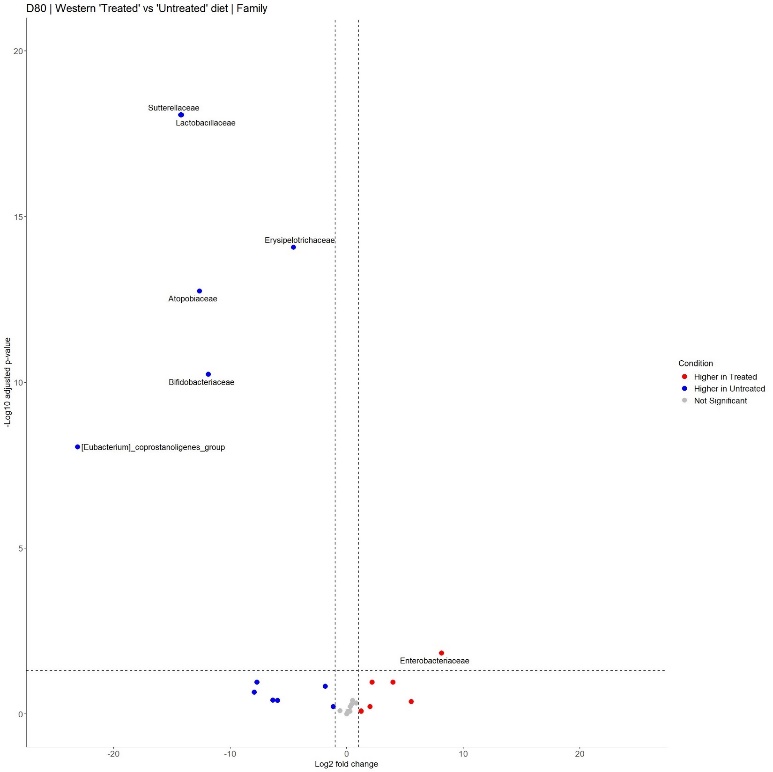

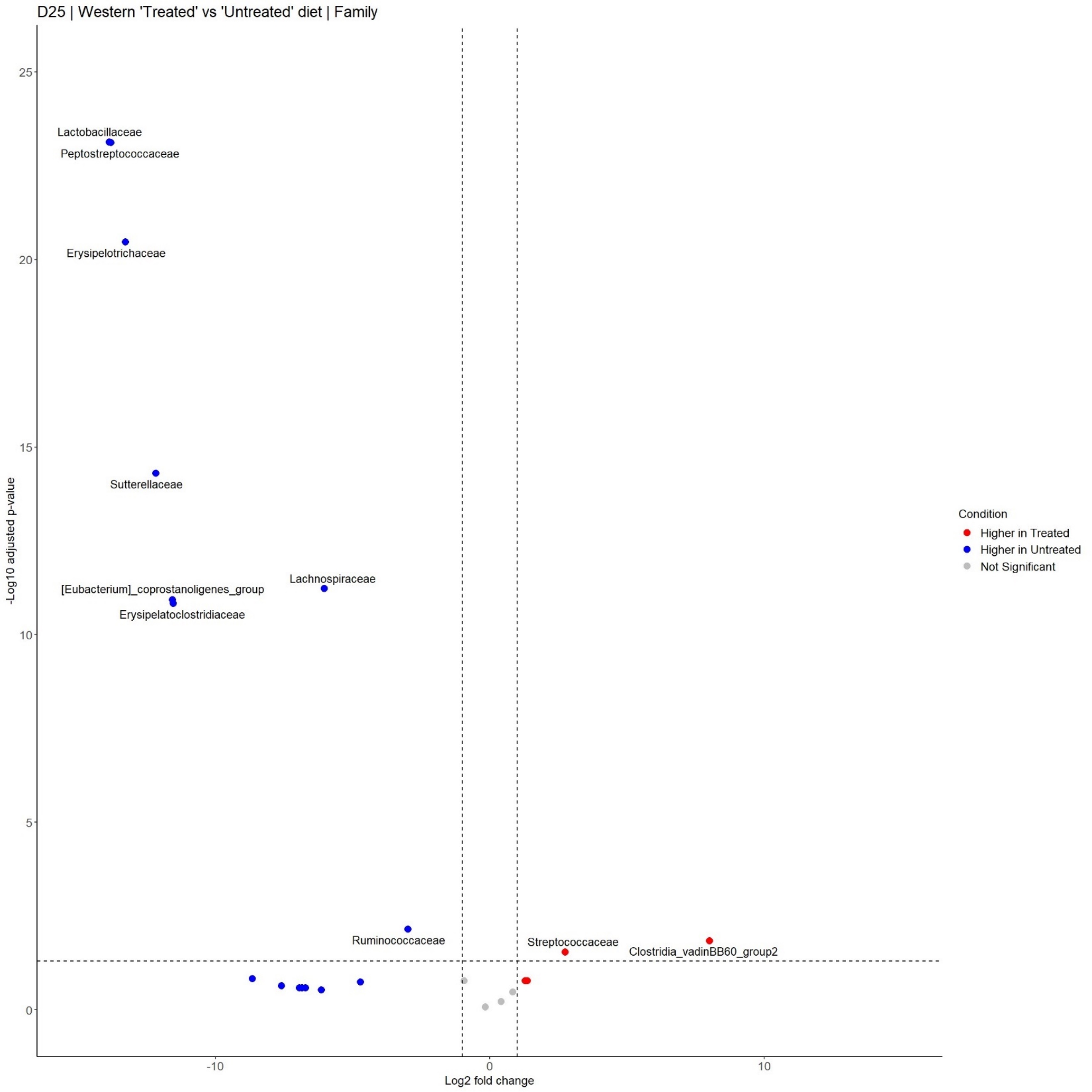


**Supplementary Figure S4 –** Family-level DESeq2 of day 25 and day 80 FJ, Western.
