# Supplementary figures and images for: Mouse diet and vendor impact microbiome perturbation and recovery from early-life pulses of amoxicillin
Source: Front Microbiomes. 2024 Jul 29;3:1432202. doi: 10.3389/frmbi.2024.1432202 (PMC12993551; doi:10.3389/frmbi.2024.1432202)

**Supplementary Data Sheet 5 – Weights by week in FJ, MJ, FC mice**


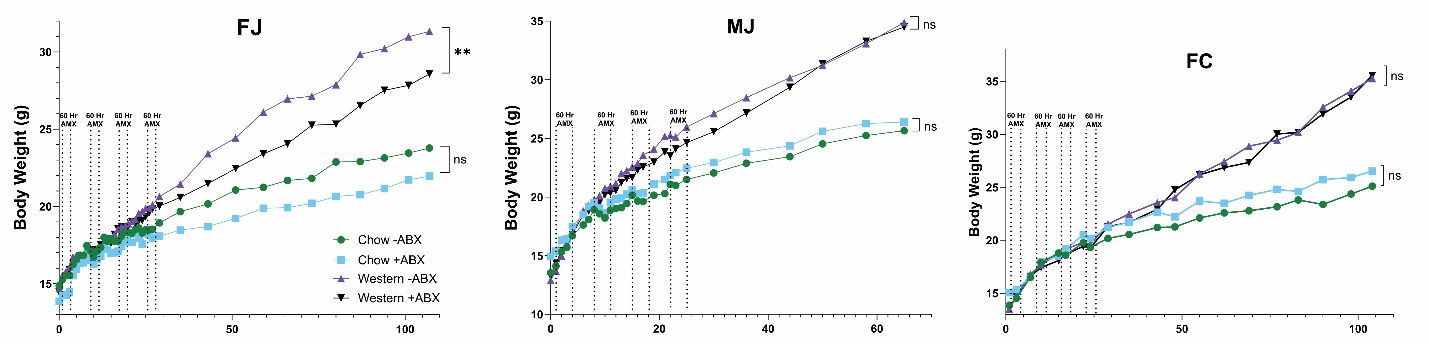

Supplement: Supplementary file 5 [file DataSheet_5.docx]
