# Supplementary material for: Mouse diet and vendor impact microbiome perturbation and recovery from early-life pulses of amoxicillin
Source: Front Microbiomes. 2024 Jul 29;3:1432202. doi: 10.3389/frmbi.2024.1432202 (PMC12993551; doi:10.3389/frmbi.2024.1432202)
Supplement: Supplementary file 6 [file Table_1.docx]

**Supplementary Table 1**

| Mouse Cohort | Comparison of Days | p-value |
| --- | --- | --- |
| FJ – Chow -abx | 2 to 4 | 0.281 |
|  | 2 to 23 | 0.266 |
|  | 2 to 25 | 0.173 |
|  | 2 to 51 | 0.012 * |
|  | 2 to 80 | 0.249 |
|  | 2 to 107 | 0.183 |
| MJ – Chow -abx | 2 to 4 | 0.007 ** |
|  | 2 to 23 | 0.011 * |
|  | 2 to 25 | 0.008 ** |
|  | 2 to 51 | 0.004 ** |
|  | 2 to 65 | 0.01 ** |
| FC – Chow -abx | 2 to 4 | 0.0546 |
|  | 2 to 23 | 0.091 . |
|  | 2 to 25 | 0.1 |
|  | 2 to 51 | 0.042 * |
|  | 2 to 80 | 0.021 * |
|  | 2 to 107 | 0.077 . |
| FJ, MJ, FC (Chow-abx) | All | .001 *** |
| FJ, FC | All | .001 *** |
| FJ, MJ | All | .001 *** |
| FJ – Western -abx | 2 to 25 | 0.064 . |
| MJ – Western -abx | 2 to 25 | 0.005 ** |
| FC – Western -abx | 2 to 25 | 0.006 ** |

**Table S1 –** PERMANOVA p-values.
