# Supplementary material for: Mouse diet and vendor impact microbiome perturbation and recovery from early-life pulses of amoxicillin
Source: Front Microbiomes. 2024 Jul 29;3:1432202. doi: 10.3389/frmbi.2024.1432202 (PMC12993551; doi:10.3389/frmbi.2024.1432202)
Supplement: Supplementary file 8 [file Table_3.docx]

**Supplementary Table 3 – MaAsLin2 values**

| Comparison | Family | p-adj |
| --- | --- | --- |
| FJ Chow+abx D4 v. FJ Chow-abx D4 | Akkermansiaceae | .0169 |
| FJ Chow+abx D25 v. FJ Chow-abx D25 | Akkermansiaceae | .0121 |
| MJ Chow+abx D4 v. MJ Chow-abx D4 | Akkermansiaceae | .3843 |
| MJ Chow+abx D25 v. MJ Chow-abx D25 | Akkermansiaceae | .0048 |
| FC Chow+abx D4 v. FC Chow-abx D4 | Akkermansiaceae | .7543 |
| FC Chow+abx D25 v. FC Chow-abx D25 | Akkermansiaceae | .0125 |
| FJ Western+abx D4 v. FJ Chow+abx D4 | Akkermansiaceae | .0057 |
| FJ Western+abx D25 v. FJ Chow+abx D25 | Akkermansiaceae | .0020 |
| MJ Western+abx D4 v. MJ Chow+abx D4 | Akkermansiaceae | .0001 |
| MJ Western+abx D25 v. MJ Chow+abx D25 | Akkermansiaceae | .0000 |
| FC Western+abx D4 v. FC Chow+abx D4 | Akkermansiaceae | .0001 |
| FC Western+abx D25 v. FC Chow+abx D25 | Akkermansiaceae | .0000 |
| FC; no abx; 4-107; Western v Chow | Anaerovoracaceae | 0.0020 |
| FC; no abx; 4-107; Western v Chow | Lactobacillaceae | 0.0006 |
| JAX (FJ and MJ); no abx; 4-65-107; Western v Chow | Lachnospiraceae | 0.0031 |
| Western (FJ & FC); no abx; 4-107; Jax vs CRL | Erysipelotrichaceae | 0.0000 |
| Western (FJ & FC); no abx; 4-107; Jax vs CRL | Atopobiaceae | 0.0000 |
| Western (FJ & FC); no abx; 4-107; Jax vs CRL | Akkermansiaceae | 0.0003 |
| Western (FJ & FC); abx; 4-107; Jax vs CRL | Bifidobacteriaceae | 0.0000 |
| Western (FJ & FC); abx; 4-107; Jax vs CRL | Clostridia_UCG.014 | 0.0001 |
| Western (FJ & FC); abx; 4-107; Jax vs CRL | Clostridiaceae | 0.0001 |
| all Western and Chow, abx v no abx | Enterobacteriaceae | 0.0000 |
| all Western and Chow, abx v no abx | Acholeplasmataceae | 0.0004 |
| all Western and Chow, abx v no abx | Clostridia_vadinBB60_group2 | 0.0027 |
| All. Abx only. Western v Chow | Enterobacteriaceae | 0.0000 |
| All. Abx only. Western v Chow | Streptococcaceae | 0.0000 |
| All. Abx only. Western v Chow | Peptostreptococcaceae | 0.0000 |

**Supplementary Table 2 –** Maaslin p-adj values.
