# Supplementary material for: Mouse diet and vendor impact microbiome perturbation and recovery from early-life pulses of amoxicillin
Source: Front Microbiomes. 2024 Jul 29;3:1432202. doi: 10.3389/frmbi.2024.1432202 (PMC12993551; doi:10.3389/frmbi.2024.1432202)
Supplement: Supplementary file 9 [file Table_4.docx]

**Supplementary Table 4 – Effect Sizes and p-values**

| Mouse Cohort | Diet – Day | Effect Size | p-value |
| --- | --- | --- | --- |
| FJ | Chow – Day 2 | 0.1279 | 0.365 |
|  | Chow – Day 4 | 0.33723 | 0.06 . |
|  | Chow – Day 23 | 0.11379 | 0.498 |
|  | Chow – Day 25 | 0.56606 | 0.037 * |
|  | Chow – Day 51 | 0.45781 | 0.011 * |
|  | Chow – Day 80 | 0.17426 | 0.382 |
|  | Chow – Day 107 | 0.22816 | 0.053 . |
|  | Western – Day 2 | 0.1589 | 0.543 |
|  | Western – Day 4 | 0.73657 | 0.06667 . |
|  | Western – Day 23 | 0.35872 | 0.009 ** |
|  | Western – Day 25 | 0.66354 | 0.009 ** |
|  | Western – Day 51 | 0.39153 | 0.01 ** |
|  | Western – Day 80 | 0.49892 | 0.003 ** |
|  | Western – Day 107 | 0.28946 | 0.049 * |
| MJ | Chow – Day 2 | 0.14391 | 0.286 |
|  | Chow – Day 4 | 0.44237 | 0.005 ** |
|  | Chow – Day 23 | 0.63183 | 0.007 ** |
|  | Chow – Day 25 | 0.8122 | 0.007 ** |
|  | Chow – Day 51 | 0.71802 | 0.021 * |
|  | Chow – Day 65 | 0.50437 | 0.01 ** |
|  | Western – Day 2 | 0.09702 | 0.548 |
|  | Western – Day 4 | 0.81756 | 0.008 ** |
|  | Western – Day 23 | 0.44475 | 0.013 * |
|  | Western – Day 25 | 0.82882 | 0.011 * |
|  | Western – Day 51 | 0.64397 | 0.027 * |
|  | Western – Day 65 | 0.22036 | 0.169 |
| FC | Chow – Day 2 | 0.08261 | 0.859 |
|  | Chow – Day 4 | 0.38802 | 0.1667 |
|  | Chow – Day 23 | 0.13971 | 0.6667 |
|  | Chow – Day 25 | 0.82098 | 0.015 * |
|  | Chow – Day 51 | 0.36686 | 0.025 * |
|  | Chow – Day 80 | 0.41648 | 0.01 ** |
|  | Chow – Day 107 | 0.44686 | 0.013 * |
|  | Western – Day 2 | 0.12088 | 0.367 |
|  | Western – Day 4 | 0.61861 | 0.029 * |
|  | Western – Day 23 | 0.28593 | 0.146 |
|  | Western – Day 25 | 0.56491 | 0.008 ** |
|  | Western – Day 51 | 0.40055 | 0.03 * |
|  | Western – Day 80 | 0.48073 | 0.013 * |
|  | Western – Day 107 | 0.26722 | 0.013 * |

Significance values as follows: ns p ≥ 0.05, * p = 0.01 to 0.05, ** p = 0.001 to 0.01, *** p = 0.0001 to 0.001, **** p < 0.0001
